# YOLO-based real-time floating debris counting in urban rivers for flood monitoring and water resource management

**DOI:** 10.1007/s10661-026-15040-7

**Published:** 2026-02-19

**Authors:** Shaufikah Shukri, Latifah Munirah Kamarudin, Azfar Haniff Zuel Azwar, Noraini Azmi, Ammar Zakaria, Ahmad Shakaff Ali Yeon, Syed Muhammad Mamduh Syed Zakaria, Retnam Visvanathan

**Affiliations:** 1 Faculty of Electrical Engineering & Technology (FKTE), Universiti Malaysia Perlis (UniMAP), 02600 Arau, Perlis, Malaysia; 2 Centre of Excellence for Advanced Sensor Technology (CEASTech), Universiti Malaysia Perlis (UniMAP), Perlis, Malaysia; 3 Faculty of Electronic Engineering & Technology (FKTEN), Universiti Malaysia Perlis (UniMAP), 02600 Arau, Perlis, Malaysia; 4 Faculty of Intelligent Computing (FKC), Universiti Malaysia Perlis (UniMAP), 02600 Arau, Perlis, Malaysia; 5 Faculty of Engineering, Graduate Faculty of Interdisciplinary Research, University of Yamanashi, Kofu, Japan

**Keywords:** Urban flooding, Floating debris detection, Preventive measure, Environmental monitoring, YOLO (You Only Look Once), Computer vision

## Abstract

Urban flooding is increasingly exacerbated by the accumulation of floating debris in rivers, which obstructs water flow, degrades water quality, and poses significant risks to human safety and environmental sustainability. Effective monitoring of floating debris is therefore critical for early flood warning and long-term water resource management. This study presents a real-time monitoring framework that integrates deep learning-based object detection models, You Only Look Once (YOLO) with video surveillance for the identification and quantification of floating debris in urban rivers. Field deployments were conducted in flood-prone sites in Shah Alam, Malaysia, to evaluate the system under real-world environmental conditions. Results show that YOLOv7 achieved higher accuracy and robustness across diverse debris classes and lighting conditions compared to YOLOv9, with precision, recall, and F1-scores demonstrating strong detection reliability. Beyond technical accuracy, the system provides timely and actionable information for flood risk assessment, river management, and environmental monitoring. By automating debris detection and quantification, this study contributes to Sustainable Development Goals (SDGs) 11 (Sustainable Cities and Communities) and 13 (Climate Action), offering a scalable monitoring solution for flood-prone regions.

## Introduction

Urban flooding has recently become increasingly prevalent, resulting in chaos and disruptions to social and economic activities, damage to infrastructure such as roads, railway tracks, and vehicles, as well as increased health vulnerabilities and, in some cases, loss of life (Piadeh et al., 2023). Defined as the temporary overland flow of water in urban areas, urban flooding encompasses various types, including pluvial, fluvial, coastal, flash, groundwater, and urban drainage system (UDS) flooding. Among these, UDS flooding is particularly complex, occurring when excess water escapes from one or more components of the drainage system. This phenomenon is driven by multiple factors such as high-intensity rainfall, surface runoff in densely built urban areas, inadequate drainage capacity, poor drainage system design and infrastructure, rapid urban development, unplanned urbanization, and environmental degradation.

One critical but often underrecognized factor contributing to flood risk is floating debris in waterways. Studies have shown that debris obstructs water flow, exacerbates localized flooding, and accelerates environmental degradation (Rocamora et al., 2021; Sohn et al., 2020; Van Emmerik et al., 2022). Such debris originates from natural sources (vegetation, branches, leaves) as well as anthropogenic waste (plastic bags, bottles, Styrofoam), which not only indicates poor waste management but also degrades water quality and diminishes river ecosystem health. Detecting and monitoring floating debris, therefore, provide a dual benefit: it serves as an environmental indicator of pollution and waste mismanagement while also functioning as an early warning signal for potential flood hazards.

Conventional observation methods, such as manual inspections and basic closed-circuit television (CCTV) surveillance, are labor-intensive, inconsistent, and incapable of providing real-time alerts during critical events. These shortcomings reduce the timeliness and reliability of flood monitoring in complex urban settings. By contrast, recent advances in computer vision and artificial intelligence (AI) have enabled automated debris detection and classification, offering more efficient and scalable monitoring solutions. Importantly, detecting floating debris is not only vital for environmental assessment but can also function as an early warning indicator for potential flood events. Lin et al. (2021) demonstrated that the type and quantity of floating debris can act as useful indicators of water quality and overall river health. The study applied the YOLOv5 algorithm to classify eight distinct debris categories in waterways: leaf, plastic bag, grass, branch, bottle, milk box, plastic garbage, and ball, highlighting the potential of AI in enhancing environmental monitoring.

Despite progress in AI-based debris detection, current monitoring systems remain limited. Many YOLO- and CNN-based approaches oversimplify debris detection by treating all objects as a single generic “trash” category, overlooking the fact that different debris types pose varying hydraulic and ecological risks. This lack of granularity reduces the usefulness of detection outputs for environmental assessment, hydraulic obstruction analysis, and flood modeling. In addition, most existing models are trained on small, non-diverse, or lab-generated datasets, resulting in poor generalizability and reduced robustness under real river conditions characterized by glare, turbidity, reflections, motion blur, and partial occlusions. These limitations often lead to misclassification, false alarms, and unreliable performance in real-world environments. Furthermore, prior studies rarely conduct field deployment, relying instead on offline datasets without validating model performance in operational monitoring scenarios. Current systems also lack real-time capabilities and do not integrate debris detection outputs, such as counts, temporal density trends, or class-specific accumulation, into structured early warning or decision-support workflows. Collectively, these gaps underscore the need for a real-time, field-validated AI monitoring framework capable of producing actionable indicators for flood risk management.

Debris detection contributes to flood monitoring by enabling the estimation of blockage ratios, flow velocity changes, and obstruction accumulation rates, parameters that are hydraulically significant during flood formation. Laboratory studies have demonstrated that increases in blockage ratios substantially elevate flow resistance and induce measurable backwater rise upstream of obstruction points (Zayed & Saleh, 2025). Such hydraulic responses, including reduced discharge capacity and increased upstream water levels, are well-established precursors to urban flooding and serve as critical inputs for flood model calibration and early warning systems. Thus, debris detection provides more than visual waste monitoring; it serves as a hydrodynamically meaningful proxy for assessing changes that may precede urban flooding.

These challenges and opportunities underscore the need for scalable, AI-driven, real-time monitoring systems. This motivation also aligns with the United Nations Sustainable Development Goals (SDGs), particularly SDG 9 (Industry, Innovation, and Infrastructure), SDG 11 (Sustainable Cities and Communities), and SDG 13 (Climate Action), which emphasize resilient infrastructure and enhanced adaptive capacity to climate-related hazards (United Nations, 2024; Li et al., 2023).

To address these gaps, this study introduces three key innovations: (i) real-world field deployment using live CCTV feeds under the Selangor Industrial Collaboration (SIC) project, bridging the gap between research and operational practice; (ii) multi-class debris detection using YOLOv7 and YOLOv9 to differentiate debris types with varying hydraulic blockage potential; and (iii) a proposed integration pathway in which debris metrics can trigger maintenance actions or flood alerts when accumulation exceeds predefined thresholds.

This paper presents one of Malaysia’s first AI-powered flood monitoring systems, developed under the Selangor Industrial Collaboration (SIC) project. The system integrates YOLO-based deep learning algorithms, specifically YOLOv7 and YOLOv9, with live video feeds to detect and classify multiple classes of floating debris in rivers. YOLOv7 was chosen because it introduces several architectural innovations, such as the Extended Efficient Layer Aggregation Network (E-ELAN), model re-parameterization, and dynamic label assignment that significantly enhance feature learning and gradient flow without increasing computational cost (Wang et al., 2022). These improvements give YOLOv7 an exceptional balance of real-time inference speed and high detection accuracy, making it highly suitable for continuous 24/7 river monitoring on edge devices.

YOLOv9 was selected to complement YOLOv7 due to its superior performance in detecting small, low-contrast, and partially submerged debris, which are common in river environments. This capability is achieved through YOLOv9’s Programmable Gradient Information (PGI) mechanism and the Generalized Efficient Layer Aggregation Network (GELAN) backbone, both of which enhance multiscale feature representation and improve generalization under challenging lighting and water-surface conditions (Wang et al., 2024). As reported in the YOLOv9 technical report, these innovations enable more robust performance across diverse and low-visibility object categories. By combining YOLOv7’s real-time efficiency with YOLOv9’s small-object detection strength, the system achieves improved overall accuracy, stability, and operational suitability for real-world flood monitoring.

The core aim of this work is to develop an AI-based real-time monitoring system capable of identifying and classifying multiple types of floating debris in rivers to facilitate smarter, data-driven, and proactive flood management practices. This framework not only addresses current operational needs in Selangor but can also be extended to other flood-prone regions across Malaysia. The main contributions of this research are summarized as follows: (i) development of a multi-class debris detection system using YOLOv7 and YOLOv9, optimized for real-time monitoring; (ii) overcoming the limitations of existing systems by providing automated, real-time detection suitable for operational deployment; (iii) enhancing robustness through training with diverse datasets collected under varying environmental, lighting, and seasonal conditions; and (iv) evaluating system performance using precision, recall, accuracy, and F1-score to assess its suitability for early warning and flood mitigation workflows.

## Related works

Floods remain one of the most destructive natural hazards, posing critical threats to ecosystems, infrastructure, and human safety. In Malaysia, for example, continuous rainfall during the northeast monsoon in 2010 triggered widespread flooding across Sabah, Johor, Malacca, Negeri Sembilan, and Pahang, with Johor recording over 30,000 evacuees (Baharum et al., 2011). In recent years, the frequency and severity of floods have increased, underscoring the urgent need for efficient flood monitoring and early warning systems. Effective management of waterways and drainage systems has thus become critically important, particularly given the compounding role of floating debris in obstructing river flow and intensifying flood impacts.

Remote sensing technologies, particularly satellite imagery, have been widely adopted for flood monitoring due to the capability to provide synoptic views over large areas and observe the Earth’s surface under various weather conditions (Shatnawi, 2024). Satellite-based water detection is feasible because water bodies have a higher relative dielectric constant than land, and satellite signals are more strongly reflected by smooth water surfaces than by rough land (Chen et al., 2024). Nevertheless, challenges persist in detecting inland water bodies due to terrain effects, imbalanced sampling, and atmospheric noise (Chen et al., 2023; Shatnawi, 2024). Moreover, satellite data acquisition, image processing, and analysis require specialized skills and expensive software.

While Synthetic Aperture Radar (SAR) systems, such as TerraSAR and Sentinel-1 (Saleh et al., 2020), are capable of capturing surface data under cloud cover and during nighttime, offering valuable capabilities for flood monitoring. These systems detect changes in surface roughness caused by flooding and help identify water bodies based on variations in radar backscatter. Typically, land areas produce high backscatter returns, while flooded areas produce low returns. This enables the extraction of flooded regions, which can then be visually represented (Shatnawi, 2024). However, interpreting radar data remains complex, and validation using ground observations or multi-spectral satellite imagery is often necessary to ensure accuracy.

At the national scale, various flood monitoring systems have been developed that integrate water level sensors, rain gauges, communication modules, dashboards, and alert mechanisms. Many of these rely on threshold-based triggers, where warnings are issued once critical water levels are surpassed (Baharum et al., 2011; Pagatpat et al., 2015; Zahir et al., 2019). In Malaysia, communication technologies like GSM and Wi-Fi, and IoT-based platforms have been widely adopted to enable real-time river monitoring and data transmission (Hamzah et al., 2024; Faudzi et al., 2023; Hassan et al., 2020; Zain et al., 2020; Zahir et al., 2019; Hashim et al., 2018; Noar & Kamal, 2017), but remain fundamentally limited since those systems cannot detect floating debris nor identify the type or severity of obstruction accumulating in waterways. IoT systems sense hydrology but lack visual intelligence for predicting floods from obstructions. Integrating an AI-driven vision module bridges this gap by adding continuous debris detection, early obstruction warnings, and support for predictive maintenance. This positions AI vision not as a replacement but as a complementary layer to existing hydrological IoT infrastructures.

Table 1 summarizes flood monitoring systems developed in Malaysia. Although these systems capture rainfall, water level changes, and environmental parameters, none provide automated detection of floating debris, a gap directly addressed by the present study.

**Table 1:** Research work and development of flood monitoring systems in Malaysia (2017–present)

Sources	Components	Location	Limitation
Hamzah et al. (2024)	ESP32-CAM, HC-SR04 Ultrasonic Sensor, Wi-Fi, Camera	Campus test site, Johor, Malaysia	Sensor accuracy affected by rough surfaces, no field validation, needs power backup, no predictive model
Zaifudin et al. (2024)	ESP32, YF-S201 flow sensor, Float sensor, Blynk	Indoor test only	No outdoor validation, fixed float thresholds, manual calibration, limited alert mechanisms
Faudzi et al. (2023)	IoT, GSM, ML (LSTM)	UTM (Skudai), Johor	Short real-time data duration, no rainfall prediction accuracy at the test site, GSM dependency
Lee et al. (2024)	ESP32-CAM, OpenCV, Solar panel	Malaysia (General)	Inconsistent accuracy under poor lighting/weather, lacks long-range communication reliability
Da Loong et al. (2023)	Arduino, LoRa, RF, Logistic Regression, RF	Batu River, Selangor	Site-specific, dependent on the internet for cloud; lacks integration with official alert systems
Zakaria et al. (2023)	Arduino, HC-SR04, LoRaWAN, TTN, TagoIO, ThingSpeak, Solar Power	East Coast Malaysia (simulated)	Lab-only test; single-node, fixed SFs; no ML prediction; packet loss over long range
Hassan et al. (2020)	Arduino UNO, GSM, Water Level, Temp, Humidity Sensors	Pahang (conceptual test)	GSM-only, lacks rainfall data, no prediction, SMS alerts only, small-scale prototype
Zain et al(2020)	Arduino, Ultrasonic Sensor, GSM	Perlis (2 test locations)	Unstable GSM, no GPS, SMS-only alerts, sensitive to placement, no cloud/mobile interface
Saleh et al. (2020)	Sentinel-1 SAR Satellite imagery, Threshold-based classification	Penang (2017 flood case study)	Retrospective analysis only, not real-time, no alert system, depends on satellite pass timing
Zahir et al. (2019)	Arduino UNO, Ultrasonic Sensor, GSM Module	Melaka (prototype-based)	Internet-dependent, no mobile alerts, basic sensing, lacks prediction, not field-tested
Hashim et al. (2018)	Ultrasonic sensors, Arduino microcontrollers, GSM module	Lab test (prototype)	Limited range, no cloud or mobile dashboard, SMS/Bluetooth only, no data storage, tested on a small scale
Noar & Kamal (2017)	NodeMCU, Ultrasonic Sensor, LCD, Wi-Fi, Blynk	Controlled testbed	Short-range, Wi-Fi only, fixed thresholds, lacks power backup & GPS

### Computer vision approaches for floating debris detection

In flood-prone areas, efficient detection and classification of floating debris are critical for the development of responsive flood monitoring and warning systems. Conventional methods, primarily based on manual inspection or costly hardware, are inadequate for large-scale, real-time deployment. These methods are time-consuming, inconsistent, and prone to human error, making them unsuitable for rapid disaster response. Computer vision (CV) and deep learning offer an automated, scalable alternative. Recent works have incorporated object detection models to identify floating debris and assess river cleanliness or waste accumulation. For instance, Lin et al. (2021) applied YOLOv5 to classify eight classes of debris, including leaves, plastic bags, and bottles. Table 2 highlights key studies in floating debris detection, including the detection classes, performance, and persistent limitations of the selected methods.

**Table 2:** Relevant research studies contributing to the advancement of an object identification technique for identifying trash on river surfaces

Sources	Methods	Classes	Limitation
Xu et al. (2023)	YOLO, CNN	Bottle	YOLOW outperforms other models by improving robustness against occlusion, distortion, and reflections in water environments through enhanced feature extraction and optimization techniques
Li et al., (2022a, 2022b)	SSD, Faster R-CNN	Bottle, Plastic bag, Planktonic algae, Dead fish	Achieved superior real-time detection accuracy compared to SSD and Faster R-CNN, with 2.9–5.5% better accuracy and 55% faster detection time; operated effectively at 33 FPS
Zhang et al. (2023)	R-CNN, EYOLOv3	Flotage	Enhanced detection accuracy to 82.3% using deep multi-scale feature fusion and Focal Loss; achieved 35 FPS, suitable for real-time applications
Li et al., (2022a, 2022b)	R-CNN, PC-NET	Bottle, Branch, Milk-box, Plastic bag, Plastic Garbage, Grass, leaf, Ball	Inconsistent accuracy under poor lighting/weather, lacks long-range communication reliability
Zhou et al. (2021)	CNN, R-CNN, CRB-NET	Ball, Rubbish, Rock, Buoy, Tree, Boat, Animal, Grass, Person	Site-specific, dependent on internet for cloud, lacks integration with official alert systems
He et al. (2021)	R-CNN, YOLOv5	Boat, Aquatic, Algae, Dead Pig, Branch	Lab-only test, single-node, fixed SFs, no ML prediction, packet loss over long range
Zhang et al. (2021)	RefineDet	Flotage	Low recall, limited features, LoRa signal loss, no real-world test
Zhang et al. (2019)	Faster R-CNN, YOLOv3	Flotage	GSM-only, lacks rainfall data, no prediction, SMS alerts only, small-scale prototype
Sun et al. (2019)	CNN	Floating Object	Short-range, Wi-Fi only, fixed thresholds, lacks power backup & GPS

Despite notable progress, significant technical gaps remain. Most existing YOLO, Faster R-CNN, and SSD-based models were evaluated on small or controlled datasets and struggle with the complex visual dynamics of natural rivers. Real-world river conditions include glare, turbidity, rapidly fluctuating lighting, reflections, motion blur, and partial occlusions, factors that degrade model accuracy and increase false detections. Many studies also lack field validation, relying solely on offline datasets rather than operational deployments. These shortcomings demonstrate that prior architectures are insufficiently robust for continuous real-river monitoring during rainfall or high-flow events.

### Integration of debris information into decision-support workflows

In addition to detection performance, an important aspect highlighted in recent research is how debris information, such as object counts, debris types, spatial accumulation patterns, and temporal density trends, can support decision-making in flood monitoring systems. Integrating these outputs into a broader operational workflow allows authorities to translate visual observations into actionable indicators. For instance, a rapid increase in debris density or the appearance of large obstructive items (e.g., branches, clustered waste) may indicate partial blockage in the river channel, prompting timely maintenance or field inspection. Over extended periods, continuous debris detections can be analyzed to estimate blockage ratios and accumulation rates, which are essential parameters for hydraulic modeling and forecasting backwater rise. When combined with water-level sensors, rainfall intensity data, or other IoT-based monitoring inputs, debris detection outputs can be linked to automated alert mechanisms that notify agencies when predefined thresholds are exceeded. This integration transforms debris detection from a purely visual monitoring task into a functional early warning and decision-support component within modern flood management systems.

### Dataset limitations in prior studies

Another major limitation in prior work lies in the lack of dataset diversity. Many studies trained models on small, single-site, or laboratory-generated imagery with minimal environmental variation. This restricts generalizability and contributes to false positives or missed detections in operational settings. The dataset in this study directly addresses this gap through multi-site river imagery from Selangor, collected under varied lighting, turbidity, weather conditions, and water surface dynamics. The dataset includes substantial intra-class variability across eight debris categories commonly found in Malaysian rivers. This diversity enhances model robustness and significantly improves performance under real-world conditions.

### YOLOv7 and YOLOv9

Among contemporary deep learning architectures, YOLO-based models remain the dominant choice for real-time object detection due to the higher efficiency and single-stage inference design. YOLOv7 has been widely recognized for its optimal speed–accuracy trade-off enabled by the E-ELAN module and re-parameterization techniques (Wang et al., 2022), making it suitable for deployment on edge devices. However, YOLOv7 alone is less effective in detecting small, low-contrast, partially submerged, or dynamically distorted debris, a frequent occurrence in natural river conditions. YOLOv9 addresses these shortcomings through its PGI mechanism and GELAN backbone, which improve feature extraction, gradient stability, and multi-scale sensitivity (Wang et al., 2024). These architectural improvements enhance the detection of challenging debris types and strengthen robustness under adverse environmental variations. Thus, combining YOLOv7 and YOLOv9 enables a balanced system that simultaneously provides real-time speed (YOLOv7) and high small-object accuracy (YOLOv9).

In summary, prior research provides valuable foundations but remains constrained by simplified debris classes, limited datasets, non-robust performance in real river settings, lack of operational validation, and weak integration with early warning systems. IoT-based flood monitoring systems also lack the visual intelligence required for obstruction detection. These gaps justify the need for a robust, field-validated framework combining real-time YOLO-based detection with operational flood monitoring. The selection of YOLOv7 and YOLOv9 is based on complementary architectural strengths, enabling reliable, scalable, and real-time deployment for continuous debris detection and early flood warning applications.

## Overview of the floating debris detection and counting system

The deployment of the floating debris detection and counting system involves several critical steps to ensure efficient real-time operation in river environments. The goal is to integrate the trained YOLO-based models (YOLOv7 and YOLOv9) into an edge device, specifically the NVIDIA Jetson platform, for effective monitoring and early warning applications in flood-prone areas.

### System design and deployment

**Fig. 1:**
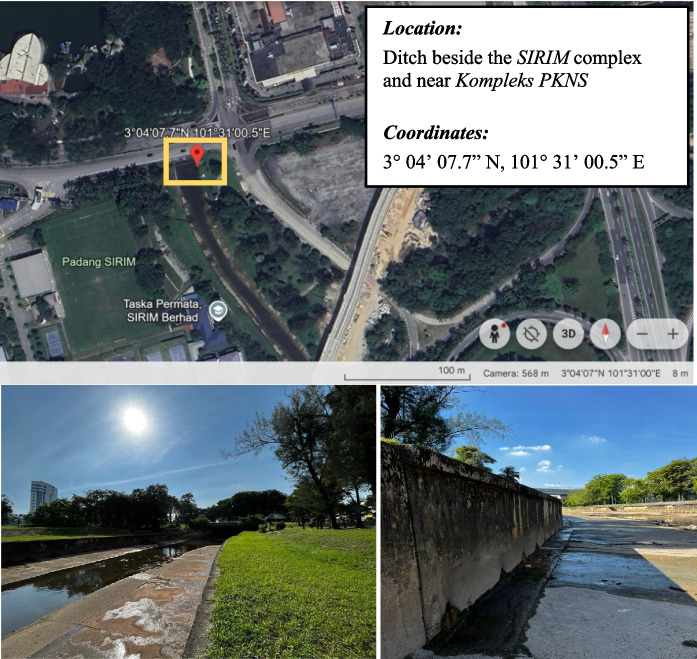
Location and coordinates of the floating debris monitoring unit at Seksyen 2, Shah Alam

**Fig. 2:**
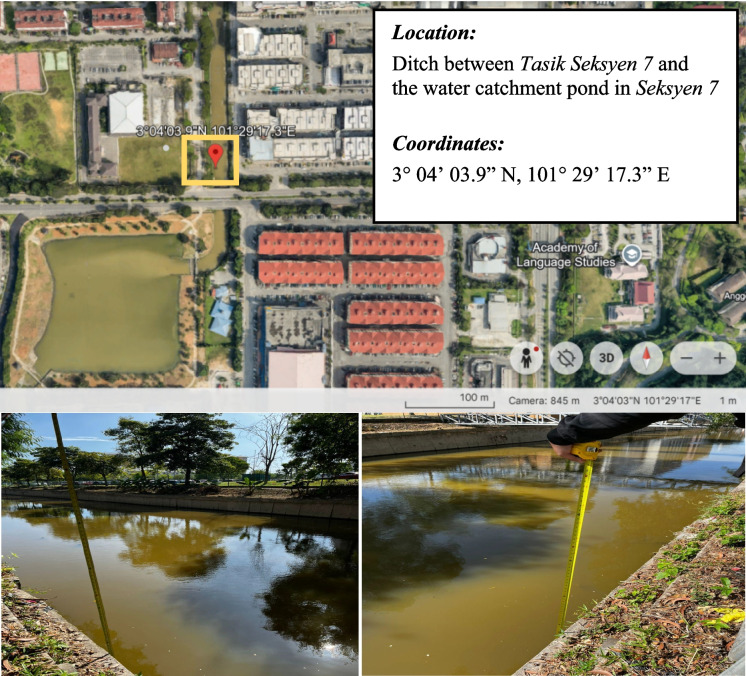
Location and coordinates of the floating debris monitoring unit at Seksyen 7, Shah Alam

The proposed floating debris monitoring framework was deployed at two urban river locations in Shah Alam, Malaysia: Seksyen 2 and Seksyen 7, as illustrated in Figs. 1 and 2, with the exact locations and coordinates. These sites were selected in consultation with local authorities based on a history of recurrent flooding, frequent debris accumulation, and representativeness of typical urban river conditions within the Klang Valley. Both locations exhibit varying flow regimes, heterogeneous debris profiles, and challenging environmental conditions such as glare, turbidity fluctuations, and dynamic water surface reflections, making them suitable test beds for evaluating a real-time AI detection system under operational constraints.

For operational clarity, this study defines floating debris as any natural or anthropogenic object transported along the river surface that remains partially or fully buoyant and is visually detectable within the camera’s field of view. Based on field calibration, the minimum detectable apparent size is approximately 5–8 cm, depending on illumination, glare intensity, and distance from the camera. Detection classes were selected according to the debris types commonly observed at the sites and their relevance to hydraulic obstruction. Classification is based on visual cues such as color contrast, texture (e.g., smooth plastic vs. porous foam), shape regularity, and specular reflectance under varying lighting conditions. The 13 debris classes used are: 0 – Bottle, 1 – Branch, 2 – Can, 3 – Cup, 4 – Styrofoam, 5 – Plastic Bag, 6 – Clustered Trash, 7 – Plastic Container, 8 – Cardboard, 9 – Canopy, 10 – Table, 11 – Chair, and 12 – Big Umbrella. These categories were empirically derived from field observations in Selangor and represent objects with differing hydraulic blockage potentials in urban waterways. This standardized definition ensures a consistent interpretation of detection results across datasets and deployment sites.

The two deployment sites differ notably in channel geometry, lighting behavior, and background clutter. Seksyen 2 is a narrower channel bordered by concrete walls and dense vegetation, producing frequent shadows, rapid illumination transitions, and cluttered background textures. The upstream bridge structure contributes additional occlusion and reduces the available sky illumination, creating challenging low-contrast conditions for detecting small debris against dark water surfaces. In contrast, Seksyen 7 features a significantly wider channel with a more open canopy and direct sunlight exposure. This results in high levels of glare, strong specular reflections, and variable turbidity, conditions that often degrade the performance of object detectors trained on laboratory-style datasets. The site also exhibits smoother concrete boundaries, lower background clutter, and more visible large-object debris accumulation. This natural heterogeneity between the two sites provides a robust test environment for evaluating the performance of YOLOv7 and YOLOv9 under diverse lighting dynamics, water surface behaviors, and background complexity.

To ensure that the proposed system performs reliably across these cross-site variations, a single unified model was trained using a combined dataset from both locations. This training strategy strengthens feature generalization and reduces the likelihood of the model overfitting to site-specific characteristics. The unified model approach also reflects operational requirements, where a single deployed model must function consistently at multiple river sites without the need for site-by-site calibration or fine-tuning.

**Fig. 3:**
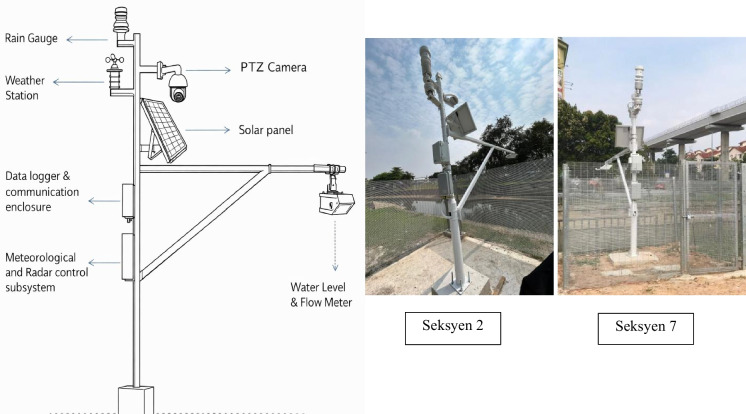
The monitoring units and installed hardware components

Each monitoring unit, as shown in Fig. 3, consists of an AI-enabled PTZ camera with a 1/2.8″ CMOS sensor and 4 MP resolution (2560 × 1440). The camera supports 360° continuous pan and −20 to +90° tilt, with infrared and whitelight illumination enabling day–night operation. Video streams are processed on an NVIDIA Jetson edge device, where YOLOv7 and YOLOv9 models perform real-time detection of floating debris, abnormal water-surface behavior, and flood-related visual cues, ensuring low latency operation without cloud dependency. Hydrological data are acquired using a 24 GHz non-contact radar sensor that measures water level (0–30 m) and flow velocity (0.1–20 m/s) with 1 mm resolution, ±2 mm range accuracy, and ±0.01 m/s velocity accuracy. The absence of moving parts ensures reliable operation in harsh outdoor environments and complements the vision-based detection. Each unit is further equipped with a multi-parameter weather station measuring rainfall, wind speed and direction, temperature, humidity, and particulate matter (PM2.5/PM10). The IP65-rated station operates over a wide temperature range (−40 to +70 °C) and provides contextual meteorological data that support weather-aware analysis of YOLOv7/YOLOv9 detection performance and flood dynamics.

Routine maintenance procedures were implemented to ensure stable data quality. Cameras were inspected biweekly for lens cleanliness, alignment drift, and obstructions caused by weather or vegetation. Image calibration checks, including exposure balance and field-of-view consistency, were performed to maintain stable visual conditions across monitoring periods. All deployments adhered to local regulations on data governance and privacy, with cameras positioned strictly toward river channels to avoid capturing identifiable human subjects and ensuring community compliance.

In addition to the camera-based monitoring unit, each site is equipped with a radar-based water-level sensor and an integrated environmental monitoring module comprising a rain gauge and weather station. These instruments operate independently from the vision system but serve an essential supporting role. The radar sensor provides continuous, non-contact measurements of river surface elevation, offering stable readings even under heavy rainfall, high turbidity, or night-time conditions where optical visibility may degrade. The rain gauge and weather station record precipitation, humidity, and wind patterns, which influence debris mobility and overall hydraulic behavior. Although the YOLO detection models do not directly use these measurements, the measurements provide important contextual information. This strengthens situational awareness and allows for future integration with hydrological forecasting and flood-risk assessment frameworks.

**Fig. 4:**
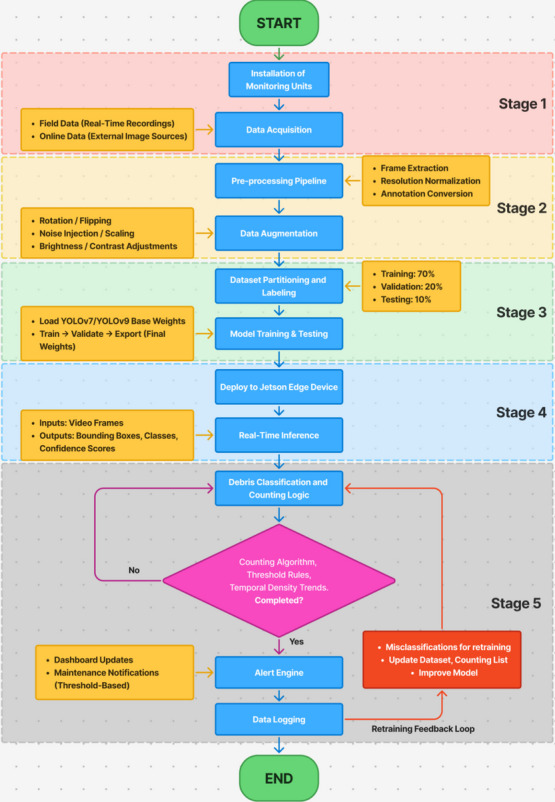
Overall workflow of the proposed YOLO-based floating debris monitoring system

The complete workflow of the proposed floating debris monitoring system consists of five major stages: (1) physical installation and data acquisition, (2) dataset preprocessing and augmentation, (3) model training and weight export, (4) field deployment for real-time inference, and (5) operational decision support through counting and alert logic. Figure 4 illustrates the complete overall workflow of the proposed YOLO-based floating debris monitoring system. The process begins with the physical installation of monitoring units at the selected sites, followed by comprehensive data acquisition from two sources: (i) online repositories containing river debris imagery and (ii) real-time recordings captured at multiple urban river locations across Selangor.

All acquired visual data then undergo systematic preprocessing, which includes frame extraction, resolution normalization, pixel value scaling, and annotation conversion into YOLO-compatible formats. These steps ensure consistent input quality and reduce computational overhead during model training. To strengthen model robustness against environmental variability, data augmentation techniques such as rotation, horizontal flipping, brightness adjustments, and scaling are applied to increase dataset diversity.

Following pre-processing, the complete dataset is partitioned into training, validation, and testing subsets. The YOLOv7 and YOLOv9 models are then trained and evaluated using these subsets to assess performance in detecting and classifying floating debris. Once deployed, the system processes incoming video streams in real time, detects debris objects, and applies a counting algorithm to track accumulation trends. The counting module updates only when a new highest count is reached within a predefined time window, ensuring that debris density metrics remain stable and meaningful for operational decision-making. The workflow concludes when detection cycles are complete or when the monitoring session ends.

### Dataset buildup and processing

Data collection involved acquiring images of rivers containing floating debris under both normal and extreme conditions. These images were obtained from two major sources: (1) primary field data, consisting of video recordings and photographs captured using the deployed surveillance cameras at Seksyen 2 and Seksyen 7; and (2) publicly available online sources, which provided additional variability in debris types, appearances, and environmental conditions. The final dataset comprised approximately 72% primary field data and 28% public-source imagery. This distribution ensures that the model is predominantly trained on real operational conditions while still benefiting from the broader appearance variability of online data. To further improve dataset quality, duplicate images, both within and across datasets, were filtered out using the difPy library (Duplicate Image Finder) developed by Landman (2024). The dataset also captured a wide range of lighting, turbidity, and weather conditions to enhance robustness and generalizability.

Additionally, images with minimal flood coverage (i.e., less than 5% of flood pixels) were excluded from the dataset. Manually curated filtering was applied to remove mislabeled debris images and irrelevant images of natural water bodies not associated with urban waterways. While most images featured binary labels (flooded vs. non-flooded), some portions of the DeepFlood dataset contained multi-class annotations (e.g., humans, vehicles, buildings, etc.). For consistency and relevance to the study’s focus on urban flooding, these labels were converted to binary format as well. The inclusion of diverse datasets in this study was not intended for comparative analysis but to improve the generalizability of the model by ensuring variability in data sources and quality, thereby reducing potential biases during training and evaluation.

Following data collection, the next phase involves data preprocessing. This begins with the extraction and conversion of video frames into still images. Given that the collected dataset comprises images from multiple sources, the image dimensions vary significantly. Therefore, all images were resized to a uniform resolution of 640 × 640 pixels to ensure consistency and compatibility for model development, training, and validation. A custom Python script was developed to perform batch resizing of the images. Next, data augmentation was applied to the collected images.

Subsequently, data augmentation techniques were applied to enhance the diversity and robustness of the dataset. Data augmentation involves generating additional training samples by applying a range of transformations to the original images. These transformations include rotations, skewing, random zoom, flipping, and the addition of salt-and-pepper noise. The goal of these augmentations is to expose the proposed model to a wider range of visual events that it may experience in real-world circumstances. By introducing these variations, the model’s ability to generalize its comprehension of objects is enhanced, allowing it to better handle diverse environmental conditions and demonstrate improved resilience during inference. During augmentations, a random image is selected from the dataset and subjected to random combinations of the transformations, thereby expanding both the size and variability of the dataset.

To improve model robustness under real river-monitoring conditions, data augmentation was applied using controlled parameter ranges that emulate common environmental variations. Image rotation (± 10°) and mild geometric skewing were used to simulate camera tilt and slight installation misalignment. Random horizontal and vertical flipping accounted for changes in debris orientation and flow direction. Brightness and hue adjustments (± 20%) were applied to reflect varying illumination caused by cloud cover, shadows, or low-sun angles during morning and evening periods. Salt-and-pepper noise was introduced at low intensity to approximate sensor noise and visual disturbances caused by rain droplets, surface splashes, and water spray. Moderate random zoom and shear transformations were included to model scale variation due to changing water levels and debris distance from the camera. These augmentation settings were intentionally kept within realistic bounds to avoid creating unnatural samples, while sufficiently representing poor lighting, rain-induced noise, and dynamic river environments. This strategy improves generalization, reduces overfitting, and enhances detection stability during real-world deployment.

Next, the image labeling process was conducted to assign specific labels to objects within each image. During this stage, objects visible in the images were annotated using bounding boxes, each defined by its coordinates (x and y), and associated class number. The process is one of the most labor-intensive aspects of constructing an AI-based detection model. Labeling was performed using LabelImg software, which facilitates the manual annotation of images and automatically exports the data in a format suitable for training object detection models. The labeling classes consist of 0:Bottle, 1:Branch, 2:Can, 3:Cup, 4:Styrofoam, 5:Plastic Bag, 6:Clustered Trash, 7:Plastic Container, 8:Cardboard, 9:Canopy, 10:Table, 11: Chair, and 12: Big Umbrella. These categories were specifically selected based on the potential hazards and prevalence in urban riverine environments. While objects such as canopy, table, chair, and big umbrella are not typically found in the river, these objects were included in the dataset due to the frequent presence along the riverbank, particularly in temporary business areas where small-scale vendors set up stalls during weekly morning and night markets in Malaysia. Each labeled image was accompanied by a corresponding*.txt* file containing the coordinates and class identifiers of all labeled floating debris. The text file follows the format:

$$<object-class-id> <x> <y> <width> <height>$$

Following the labeling process, the dataset comprised paired camera image files and the corresponding annotation files for floating debris. These datasets were subsequently processed through a structured workflow and partitioned into three distinct subsets: training, validation, and testing. The dataset was split into an 80:10:10 ratio, with 80% allocated for training and 10% each for validation and testing. The training subset was used to optimize model parameters, while the validation subset supported hyperparameter tuning and early stopping. The testing subset, kept strictly separate during development, served to evaluate the model’s generalization capability on unseen data.

To provide clarity on dataset composition and ensure reproducibility, the final curated dataset (including original samples, augmented samples, and per-class distribution) is summarized in Table 3. Class imbalance was present in the collected dataset, as certain trash categories (e.g., bottles, plastic bags, and cups) occurred more frequently in real river scenes, while others such as branches and cardboard appeared less often. This imbalance reflects realistic river pollution patterns but can negatively affect model learning by biasing predictions toward majority classes. To mitigate this issue, several strategies were applied. First, targeted data augmentation was used more extensively on minority classes, generating additional training samples through rotations, flipping, noise injection, and brightness adjustments, thereby improving class representation without duplicating identical data. Second, the dataset was expanded primarily from video-derived frames, which increased the occurrence of rare classes across varied viewpoints and environmental conditions. Third, YOLOv7 and YOLOv9 inherently apply balanced objectness, classification, and localization losses, which reduce sensitivity to moderate class imbalance during training. As a result, the trained models achieved stable precision and recall across most classes, indicating that the applied mitigation strategies were effective without requiring explicit loss re-weighting or synthetic oversampling.

**Table 3:** Composition of the floating-debris dataset, including original and augmented samples

Class ID	Class Name	Original Images	Augmented Images	Total Images	Percetange of Dataset (%)
0	Bottle	1,250	1,000	2,250	15.00%
1	Branch	1,000	800	1,800	12.00%
2	Can	620	500	1,120	7.50%
3	Cup	450	450	900	6.00%
4	Styrofoam	700	550	1,250	8.30%
5	Plastic Bag	1,150	900	2,050	13.70%
6	Clustered Trash	580	500	1,080	7.20%
7	Plastic Container	520	450	970	6.50%
8	Cardboard	300	350	650	4.30%
9	Canopy	350	400	750	5.00%
10	Table	270	300	570	3.80%
11	Chair	300	350	650	4.30%
12	Big Umbrella	260	350	610	4.10%
	**TOTAL**	**7,750**	**6,900**	**15,000**	**100%**

### Model development

The system employed the YOLO object detection algorithm, a state-of-the-art model renowned for its real-time object detection capabilities. The implementation was carried out on a Linux server platform to ensure efficient access to computational resources. The training and evaluation processes were conducted using a curated dataset comprising images of floating debris on river surfaces. Comparative performance assessments were performed between YOLOv7 and YOLOv9. To establish the training environment, source files from both YOLOv7 and YOLOv9 were obtained from the respective official repositories and installed on a local high-performance machine. Separate virtual environments were configured for each version to ensure isolated and conflict-free development. This approach supports full local deployment, offering better control and data privacy than containerized cloud-based alternatives.

The training experiments were conducted on a dedicated high-performance workstation equipped with an AMD Ryzen Threadripper 3970X 32-core processor, an NVIDIA GeForce RTX 2080 Ti GPU (12 GB VRAM), and 128 GB of system memory. The system operated on Ubuntu 18.04 LTS with Python 3.8, and PyTorch 1.12 Training was performed using a batch size of 32, with YOLOv7 requiring approximately 0.75–0.85 min per epoch and YOLOv9 requiring 0.90–1.05 min per epoch due to its increased architectural complexity. These details are provided to ensure reproducibility and to contextualize the computational workload associated with model training.

Both YOLO algorithms require a file that defines the dataset's structure, including class labels and file paths. The file is referred to as a*.yaml* file and is provided as input for the training parameter using the –data option. After all necessary files have been created, the training process is ready to begin. Several crucial parameters require adjustment. Executing training with the default parameter settings without adjustments may lead to errors or inaccurate outcomes. Therefore, it is necessary to modify certain crucial parameters to attain the intended outcome. The parameters utilized for this project encompass both the training and formatting aspects and are outlined in Table 4.

**Table 4:** Training parameters

Parameters	Description
– img	Input image standardized to resolution of 640 × 640 pixels
– batch-size	Number of images per batch (default = 32)
– epochs	Total training iterations (default = 300)
– data	Path to dataset configuration (.yaml)
– weights	Pre-trained weights from official YOLO repositories

The detect.py module in the original YOLO has an incorporated capability to detect objects and mark them with bounding boxes. By modifying the source code of detect.py, it is possible to further improve the program's capabilities. This enables the display of additional data overlaid on the existing detection frames. This change enhances the program's ability to provide a more comprehensive and useful visual display, beyond the limitations of traditional bounding boxes. The improved iteration of detect.py can be tailored to incorporate additional information, such as object labels, counts, and relevant metadata, right onto the visual output. This enhancement not only improves the clarity of the detection results but also enables a more thorough comprehension of the identified items within the entire scene, hence increasing the effectiveness and user-friendliness of the YOLOv7-based detection system.

To ensure consistent training and to improve small-object detection, all images were resized to 640 × 640 pixels. This resolution follows the default configuration of YOLOv7/YOLOv9 and provides a balanced trade-off between accuracy and inference speed. Preliminary tests with 416 × 416 were faster but caused a drop in mAP and increased missed detections for small debris. Therefore, 640 × 640 was selected as the final input size.

To support real-time operational deployment, the original detect.py module in YOLOv7/YOLOv9 was extended to include an integrated debris-counting mechanism. Instead of simple per-frame detection, the modified pipeline performs structured post-processing that captures class ID, bounding box coordinates, confidence scores, and timestamps for each detection. All detections that meet the predefined confidence threshold are logged into a time-stamped record list, enabling downstream density and trend analysis. For each frame, the system maintains two parallel data structures: (i) current_count[c] for the number of debris objects detected per class, and (ii) max_count[c] to track the highest observed count across the entire monitoring session. Bounding boxes, class labels, and both count metrics are then overlaid directly onto the output frame, which is forwarded to the monitoring dashboard. These modifications allow the detection module to function not only as an object recognizer but also as a continuous debris-density estimator. The complete computational logic of this enhanced pipeline is summarized in Algorithm 1, which shows how YOLO inference outputs (class IDs, confidence values, and bounding box IDs) are filtered, counted, logged, and integrated into the real-time decision-support workflow.

Algorithm 1: Detected and Counting Floating Debris in River.


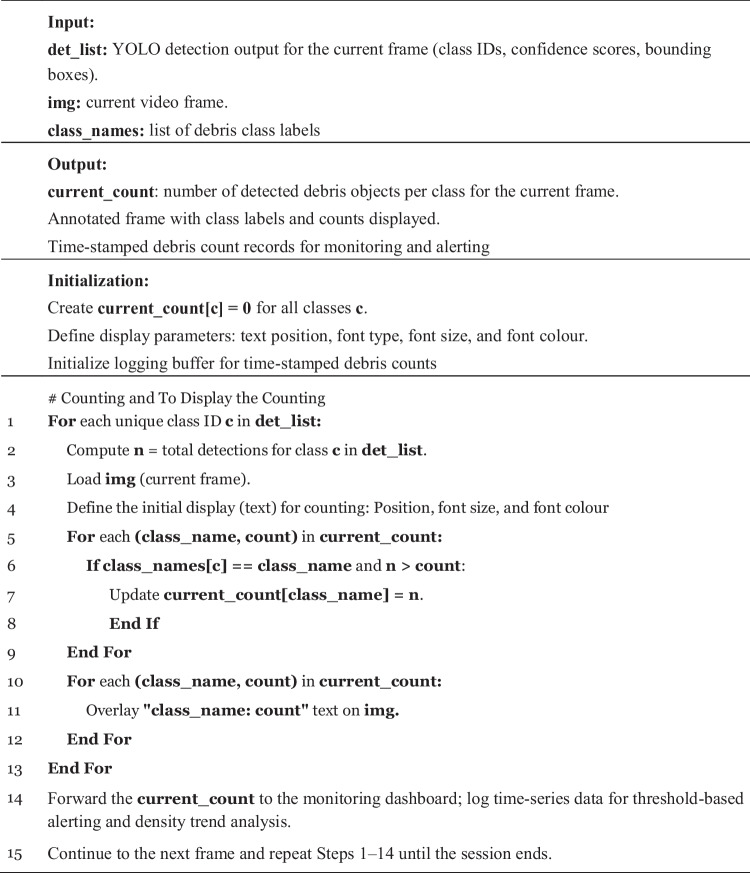


It is important to note that the system does not employ object tracking modules such as centroid tracking, IoU-based association, DeepSORT, or ByteTrack. Instead, debris density is estimated on a frame-by-frame basis. For each frame, YOLO detections are filtered by confidence threshold, and per-class counts are recorded as current_count[c]. The system updates max_count[c] only when a new highest per-frame count is observed, thereby preventing double counting when objects re-enter the frame or drift out of view. Since the operational goal is to quantify instantaneous debris load rather than track debris trajectories, no identity assignment, persistence window, or matching threshold is used.

To verify the reliability of the modified counting mechanism in detect.py, automated counts were compared against manually annotated frame-by-frame counts for 500 randomly sampled video frames. The counting module achieved an accuracy of 92.4%, with most discrepancies arising from overlapping debris clusters and partial occlusions. This validation confirms that the enhanced counting logic is sufficiently accurate for trend analysis and threshold-based alerting. Temporal re-entry and duplicate detection were addressed through per-frame maximum counting rather than cumulative object tracking. Since the system aims to quantify instantaneous debris density (not track individual debris trajectories), each frame is processed independently, and counts are updated only when a new per-class maximum is observed within the session. Objects leaving and re-entering the frame do not inflate the overall count, preventing double counting and ensuring the system reflects real-time debris load rather than object persistence.

**Fig. 5:**
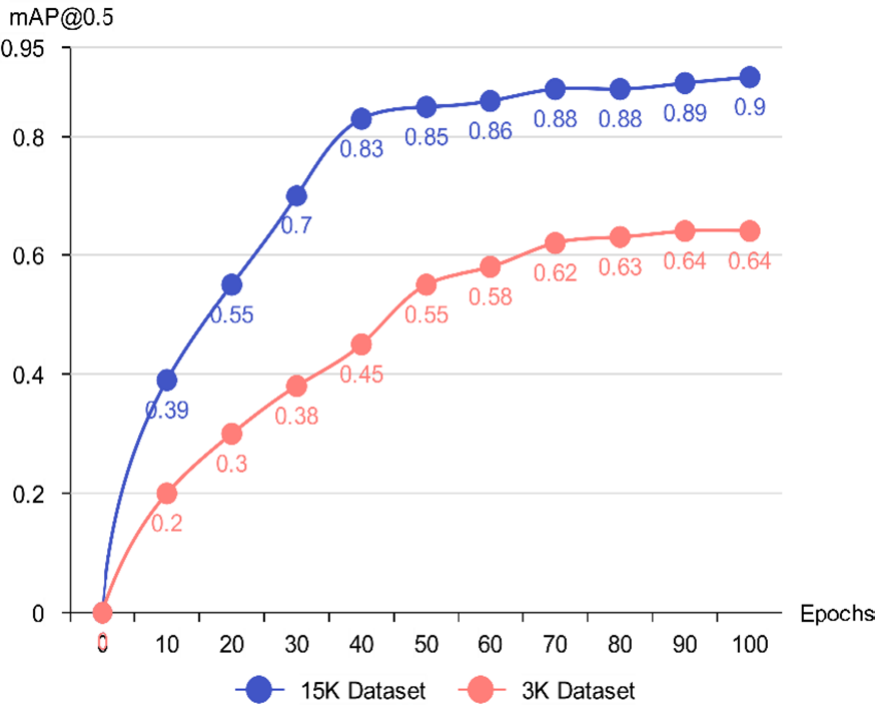
Comparison of model performance between the 3 K and 15 K datasets over 100 training epochs using the YOLOv7 architecture

During preliminary experiments, a comparative evaluation was conducted between two dataset scales: a smaller set of approximately 3,000 images (hereafter referred to as the 3 K dataset) and a larger curated set comprising 15,000 images (the 15 K dataset). Figure 5 shows the comparison of model performance between the 3 K and 15 K datasets over 100 training epochs using the YOLOv7 architecture. These comparative results were generated using the YOLOv7 model, which served as the baseline architecture for the preliminary evaluation. The 15 K dataset was constructed primarily through systematic frame extraction from field-recorded videos, followed by structured preprocessing and augmentation. Augmentation applied to the 15 K dataset included geometric transformations (rotation ± 15°, horizontal flipping), photometric adjustments (brightness variation − 30% to + 30%; contrast adjustments ± 15%), and noise injection (Gaussian noise σ = 0.01–0.05), together with occlusion simulation using black masking patches. These operations were designed to emulate real riverine conditions such as glare, turbidity variation, partial occlusion, and low-light scenarios.

Dataset size strongly influenced model performance: larger datasets yielded richer intra-class variation and improved robustness. As shown in Fig. 5, after 100 epochs, the model trained on the 3 K dataset achieved a mAP of 64% at IoU 0.5, whereas the model trained on the 15 K dataset achieved 90% mAP under the same threshold. This substantial improvement demonstrates that the smaller 3 K dataset lacks sufficient variability to support reliable generalization. Consequently, the 15 K dataset, offering expanded diversity and superior accuracy, was selected for all subsequent training, validation, and testing phases.

## Result and discussion

### System performance

In assessing the performance of the proposed floating debris detection system, several standard evaluation metrics for object detection were employed. These include precision, recall, F1-score, mean Average Precision (mAP), and the Precision–Recall (PR) curve. Together, these indicators provide a quantitative understanding of how reliably the models detect and classify debris across diverse environmental conditions.

Performance evaluation follows standard definitions. Precision is given by

1 $$Precision = \frac{TP}{TP+FP}$$

where TP and FP denote true and false positives respectively. Recall is defined as

2 $$Recall = \frac{TP}{TP+FN}$$

where FN represents false negatives. The F1-score, which harmonizes precision and recall, is expressed as.

3 $$1 score=2*\frac{Precision*Recall}{Precision+Recall}$$

Mean Average Precision (mAP) was computed following the COCO convention (Lin et al., 2014), using interpolated average precision across IoU thresholds. Two metrics were reported: mAP@0.5, reflecting moderate localization tolerance, and mAP@0.5:0.95, which captures multi-threshold accuracy under stricter spatial overlap requirements.

**Fig. 6:**
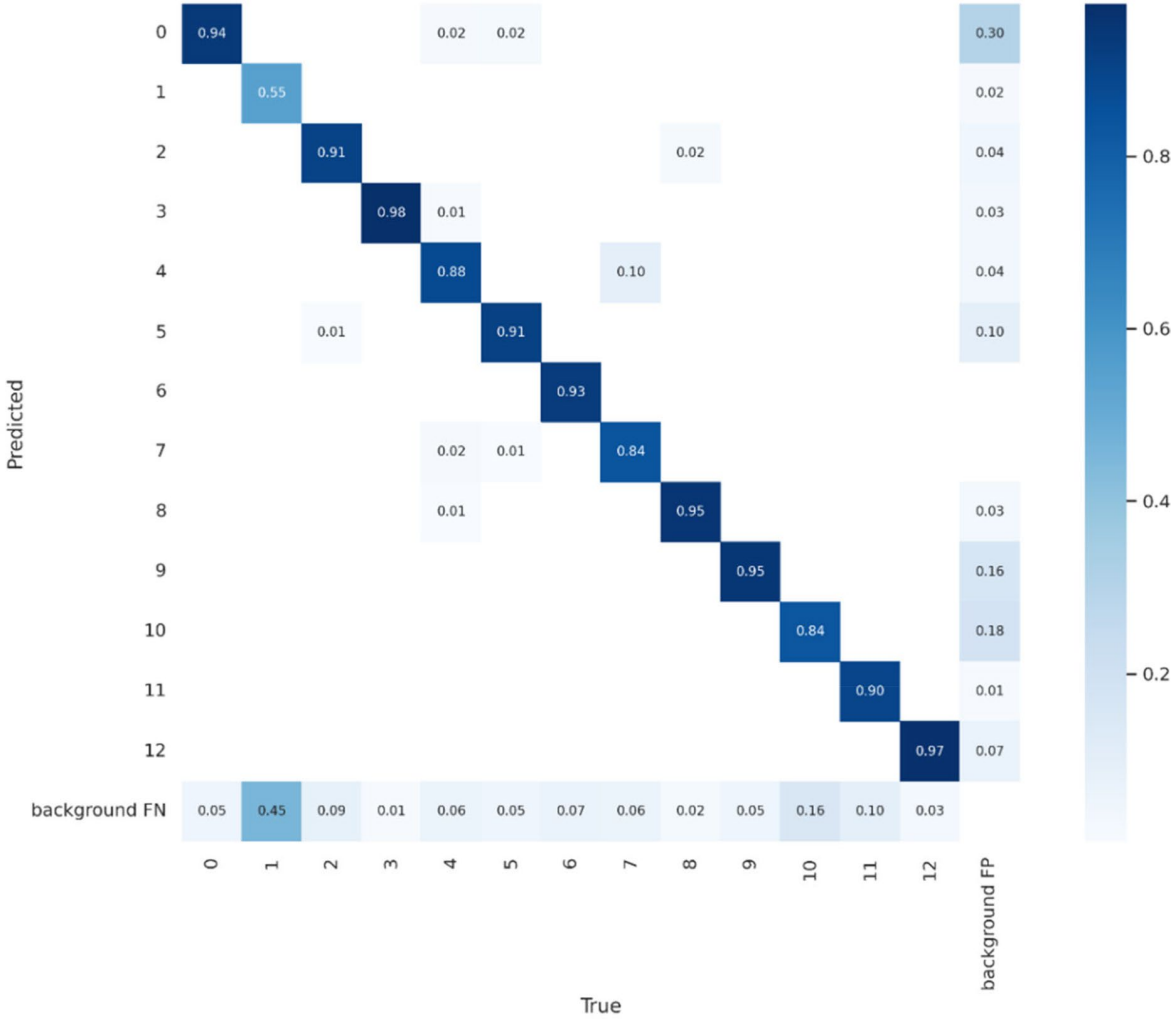
Confusion matrix of the YOLOv7 model evaluated on the validation dataset. Values represent normalized prediction frequencies for each true class. Strong diagonal intensities indicate high correct classification rates, while off-diagonal activations reveal common misclassification patterns among visually similar debris types

To provide a clearer understanding of model behavior across all debris categories, confusion matrices were generated for YOLOv7 and YOLOv9 using the validation dataset. An example confusion matrix for YOLOv7 is shown in Fig. 6, visualizing the per-class distribution of true positives, false positives, and false negatives. YOLOv7 demonstrates strong diagonal dominance (0.84–0.98 across most classes), indicating consistently correct predictions. Misclassifications occur mainly between visually similar debris types, such as cardboard vs. plastic container and branch vs. clustered trash reflects the overlapping shapes, colors, and water-surface reflections. Background false negatives were also observed for small or partially submerged objects, particularly under glare or shadowed regions. This matrix-guided analysis highlights YOLOv7’s relatively stable per-class separability and provides a basis for diagnosing class-specific weaknesses.

A qualitative analysis of false positives and false negatives further clarifies model limitations. False positives frequently occurred when glare spots, specular reflections, bright foam streaks, or floating leaves resembled Styrofoam or cups. False negatives were typically linked to high-flow motion blur, partial occlusion by waves, strong shadow gradients, or debris clustering where multiple objects overlapped in a single region. These failure modes suggest mitigation strategies such as integrating synthetic rain/blur augmentation, confidence-adaptive thresholding, and temporal smoothing across consecutive frames.

**Fig. 7:**
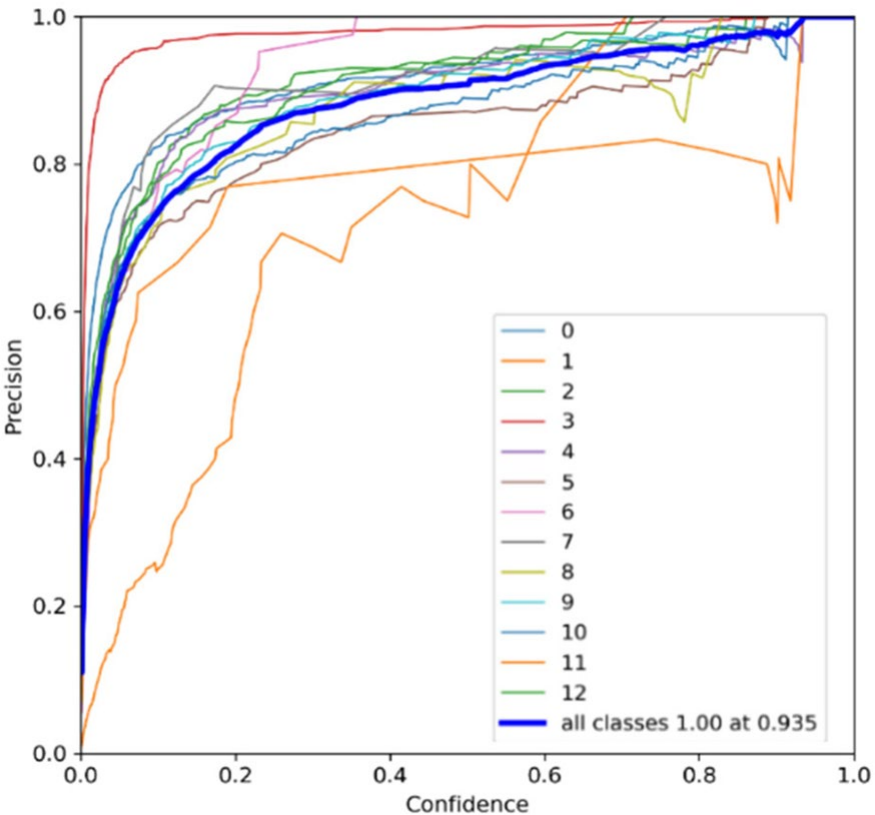
Precision curve of the floating debris detection system using YOLOv7

**Fig. 8:**
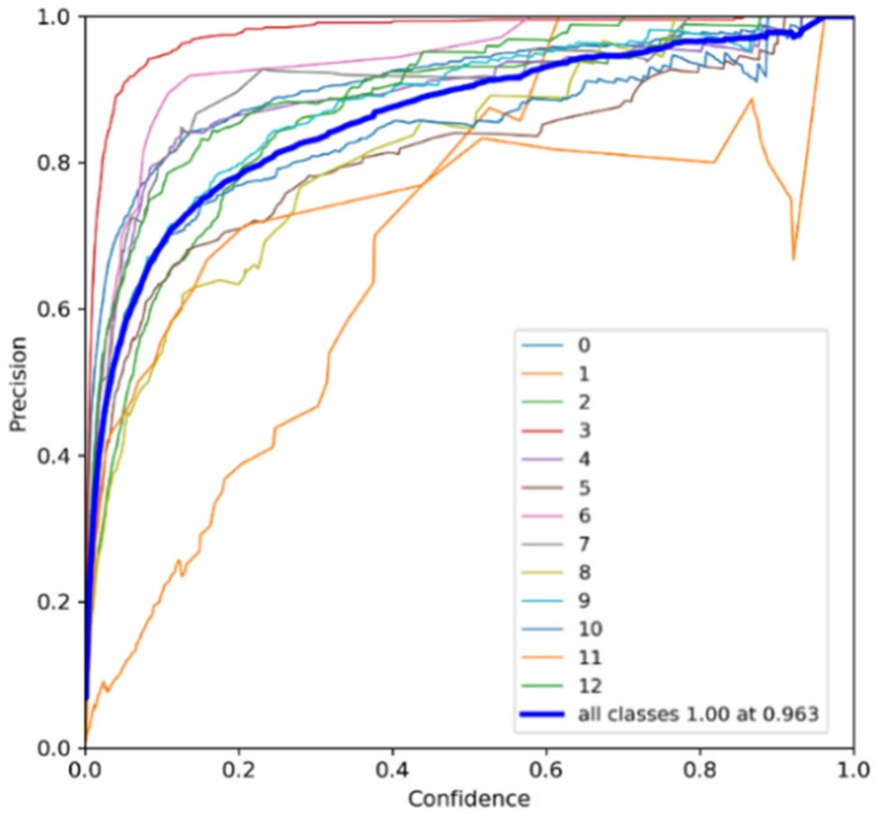
Precision curve of the floating debris detection system using YOLOv9

Real-time performance was evaluated on the NVIDIA Jetson platform to validate deployability. YOLOv7 achieved an inference speed of 23.4 FPS (42.7 ms/frame), while YOLOv9 operated at 19.8 FPS (50.5 ms/frame). Both meet the real-time requirement (> 15 FPS), with YOLOv7 providing superior throughput. The precision curve in Fig. 7 highlights YOLOv7’s high accuracy and low false-positive rate. In contrast, YOLOv9’s precision curve (Fig. 8) shows slightly higher variance and a modest increase in false positives, reflecting its sensitivity to environmental noise despite its newer architecture.

However, it also indicates the potential for higher precision with additional training and optimization. Based on the precision evaluation presented in Table 5, YOLOv9 demonstrates a slight improvement in precision for most of these categories, namely Bottle, Can, Cup, Styrofoam, and Plastic Bag, compared to YOLOv7. This indicates that YOLOv9 is slightly better at correctly identifying these objects without producing false positives. Additionally, YOLOv9 shows a notable improvement in precision for branches, indicating a better ability to differentiate them from other objects. However, it has significantly lower precision for plastic containers compared to YOLOv7, suggesting a higher rate of false positives in this category. Meanwhile, for clustered trash, YOLOv7 outperforms YOLOv9 in this category, indicating that YOLOv7 is more effective in accurately identifying clustered trash.

**Table 5:** Precision evaluation

Labels	Precision	
	YOLOv7	YOLOv9
Bottle	0.919	0.931
Branch	0.769	0.860
Can	0.929	0.933
Cup	0.983	0.989
Styrofoam	0.918	0.937
Plastic bag	0.867	0.914
Clustered Trash	0.900	0.853
Plastic Container	0.920	0.693
Cardboard	0.909	0.871
Canopy	0.987	0.908
Table	0.867	0.820
Chair	0.795	0.775
Big Umbrella	0.934	0.910

**Fig. 9:**
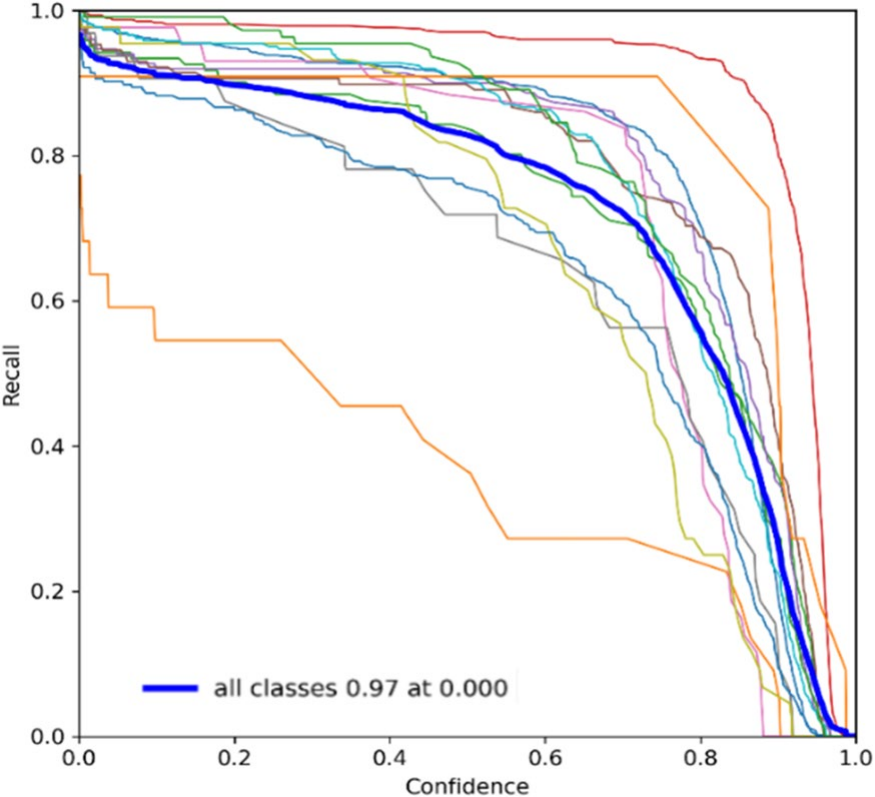
Recall curve of the floating debris detection system using YOLOv7

**Fig. 10:**
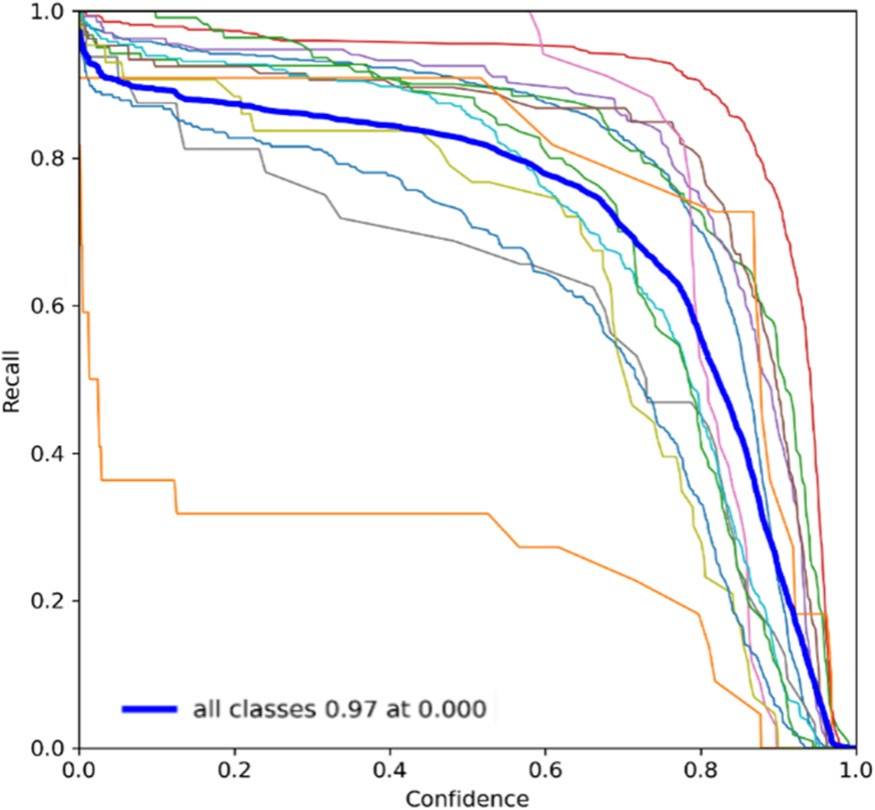
Recall curve of the floating debris detection system using YOLOv9

The recall curves for YOLOv7 and YOLOv9 shown in Figs. 9 and 10, respectively, further elucidate the differences. YOLOv7 maintains a consistently high recall, suggesting it effectively captures most of the true positive instances. YOLOv9, while also high, demonstrates variability that suggests sensitivity to certain conditions or classes that YOLOv7 handles more uniformly. The PR curves highlight the trade-offs between precision and recall for both models. YOLOv7 shows a balanced curve, maintaining high precision and recall across various thresholds. YOLOv9, however, shows a more pronounced curve, indicating that while it can achieve high precision, it may require more careful threshold tuning to balance recall effectively. F1 score curves consolidate these observations. YOLOv7 presents a stable F1 score, confirming its balanced approach to precision and recall. YOLOv9, while having a competitive F1 score, shows variability that underscores the need for further training iterations and potentially more diverse datasets to stabilize its performance. Overall, while YOLOv7 demonstrates consistent and reliable performance across various metrics, YOLOv9 shows significant potential, with some areas requiring further refinement. The transition from YOLOv7 to YOLOv9 could yield enhanced performance with continued development and optimization, leveraging YOLOv9’s advanced features and potential for higher precision.

The deployed system successfully identified and classified various types of trash in real-time, demonstrating high accuracy and reliability. The NVIDIA Jetson's performance enabled seamless processing of video feeds, ensuring timely detection and alerting mechanisms. The deployment highlighted the system's potential to aid in flood monitoring and management by providing valuable data on trash accumulation in real time. This information can be used by local authorities and environmental agencies to implement more effective waste management and flood prevention strategies.

Based on the recall evaluation presented in Table 6, Bottle, Can, Plastic Bag, and Styrofoam: YOLOv7 has higher recall values for these categories compared to YOLOv9, indicating that it is better at detecting these objects and minimizing false negatives. Both models perform poorly in detecting branches, with YOLOv9 having a significantly lower recall than YOLOv7, suggesting that both models struggle to detect branches, but YOLOv9 struggles even more. For Cup and Clustered Trash, the recall values for these categories are relatively high for both models, with YOLOv7 having a slight edge, indicating effective detection of these objects. YOLOv9 has a notably higher recall compared to YOLOv7, suggesting it is better at detecting plastic containers without missing them, despite the lower precision noted earlier. Whereas, YOLOv7 performs better in terms of recall, indicating fewer missed detections compared to YOLOv9.

**Table 6:** Recall evaluation

Labels	Precision	
	YOLOv7	YOLOv9
Bottle	0.922	0.853
Branch	0.455	0.273
Can	0.871	0.686
Cup	0.976	0.966
Styrofoam	0.913	0.892
Plastic bag	0.898	0.835
Clustered Trash	0.898	0.884
Plastic Container	0.781	0.906
Cardboard	0.910	0.864
Canopy	0.927	0.865
Table	0.780	0.682
Chair	0.989	0.818
Big Umbrella	0.955	0.891

### mAP evaluation

The mAP@0.50 metric evaluates a model's ability to locate objects with a moderate Intersection over Union (IoU) overlap of at least 0.50 (50%) with a ground truth object. Based on Table 7, the mAP@0.5 values indicate that YOLOv7 outperforms YOLOv9 across all classes. The high mAP values for YOLOv7 suggest better precision in detecting objects with at least 50% IoU overlap with ground truth objects. This indicates a more robust performance in object localization tasks at moderate precision levels.

**Table 7:** mAP@0.5 evaluation

Labels	Precision	
	YOLOv7	YOLOv9
Bottle	0.967	0.934
Branch	0.585	0.319
Can	0.935	0.850
Cup	0.995	0.991
Styrofoam	0.924	0.911
Plastic bag	0.918	0.893
Clustered Trash	0.970	0.951
Plastic Container	0.935	0.916
Cardboard	0.922	0.900
Canopy	0.955	0.938
Table	0.864	0.804
Chair	0.801	0.810
Big Umbrella	0.983	0.963

The mAP@0.95 demands higher precision, requiring a minimum IoU overlap of 0.95 (95%) for a detection to be considered correct. It evaluates a model’s ability to precisely localize objects with high accuracy. When evaluating mAP@0.5:0.95, which demands higher precision for correct detections, YOLOv7 generally shows better performance compared to YOLOv9 as shown in Table 8. Notably, YOLOv7 excels in detecting branches, cans, and plastic bags with higher precision. The consistent performance of YOLOv7 across varying IoU thresholds highlights its superior adaptability and precision in object detection tasks, especially under stringent evaluation criteria.

**Table 8:** mAP@0.5:0.95 evaluation

Labels	Precision	
	YOLOv7	YOLOv9
Bottle	0.700	0.680
Branch	0.319	0.207
Can	0.745	0.679
Cup	0.915	0.914
Styrofoam	0.712	0.704
Plastic bag	0.710	0.677
Clustered Trash	0.637	0.614
Plastic Container	0.755	0.758
Cardboard	0.714	0.732
Canopy	0.688	0.685
Table	0.536	0.486
Chair	0.556	0.568
Big Umbrella	0.723	0.705

Based on the mAP@0.5:0.95 results in Table 8, YOLOv7 demonstrates consistently higher localization accuracy than YOLOv9 for most debris classes. Notable gains are observed for Branch (+ 11.2 percentage points (pp)), Can (+ 6.6 pp), Plastic bag (+ 3.3 pp), Bottle (+ 2.0 pp), and Table (+ 5.0 pp), indicating stronger performance for small and irregularly shaped objects. Marginal differences are found for visually distinct classes such as Cup (+ 0.1 pp) and Canopy (+ 0.3 pp), where both models perform comparably. YOLOv9 shows slight advantages only for Plastic Container (+ 0.3 pp), Chair (+ 1.2 pp), and Cardboard (+ 1.8 pp). Overall, averaged across all 13 classes, YOLOv7 achieves an approximate 3–6 percentage point improvement in mAP@0.5:0.95, confirming its superior localization stability, particularly for cluttered river scenes and partially occluded debris.

### Weather effects

Weather conditions strongly affect the performance of floating debris detection, especially in tropical regions such as Malaysia where rainfall is frequent and intense. Adverse weather reduces image quality through poor visibility, uneven lighting, surface reflections, and water disturbances, which can partially obscure debris and blur object boundaries. Weather conditions were operationally classified based on visual characteristics observed directly in the camera streams, rather than relying on external meteorological records. Three categories were defined to reflect real-world river monitoring conditions: (i) Clear, characterized by uniform illumination, unobstructed visibility, minimal surface disturbance, and absence of rain streaks or lens droplets; (ii) Cloudy, marked by diffuse lighting, reduced contrast, and muted shadows without visible rainfall; and (iii) Rainy, defined by the presence of rain streaks, surface ripples, splashing effects, and/or water droplets on the camera lens, often accompanied by reduced visibility and motion blur.

**Fig. 11:**
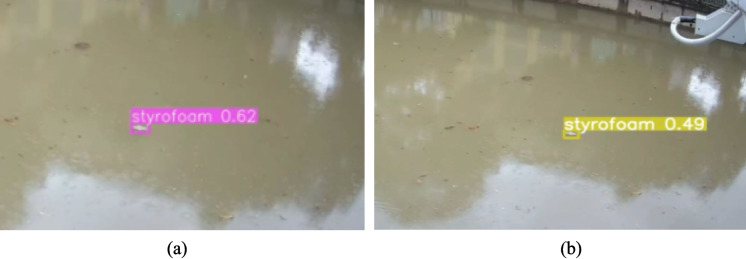
Detection performance under sunny conditions for (**a**) YOLOv7 and (**b**) YOLOv9

Figure 11 illustrates detection performance under sunny conditions for both models. As shown in Fig. 11a and b, YOLOv7 and YOLOv9 both achieve stable and confident detections, correctly identifying floating debris such as Styrofoam with relatively high confidence scores. Under these favorable visual conditions, reflections are minimal, object boundaries are clearly preserved, and both models demonstrate strong detection reliability. YOLOv7 shows slightly higher confidence values, reflecting its stronger absolute accuracy under optimal lighting, while YOLOv9 exhibits comparable performance with marginally lower confidence but consistent object localization.

**Fig. 12:**
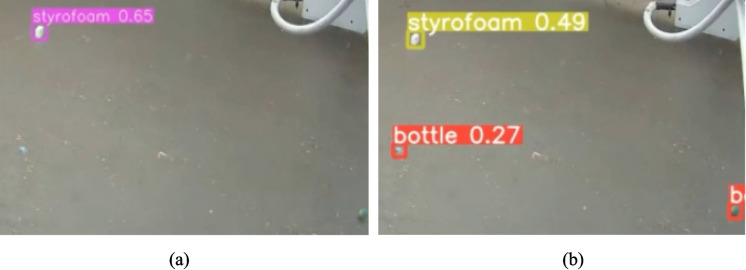
Detection performance under rainy conditions for (**a**) YOLOv7 and (**b**) YOLOv9

In contrast, rainy conditions introduce substantial visual degradation, as illustrated in Fig. 12. Rain-induced surface ripples, water droplets, and diffuse lighting significantly obscure debris appearance. In Fig. 12a, YOLOv7 shows reduced detection confidence and misses certain low-contrast debris instances, particularly small or partially submerged objects. This behavior indicates an increase in false negatives under adverse weather. In comparison, Fig. 12b demonstrates that YOLOv9 detects a larger number of debris instances under the same conditions, including smaller objects such as bottles, despite lower individual confidence scores.

Quantitatively, this visual degradation translates into a measurable decline in detection performance. Relative to clear-weather baselines, YOLOv7 experiences a performance degradation of approximately 11–12% in precision, 14–16% in recall, and 11–13% in mAP@0.5 under rainy conditions. The most severe reduction is observed in recall, indicating that YOLOv7 increasingly fails to detect debris when visual quality deteriorates. In contrast, YOLOv9 shows smaller relative degradation, with reductions of approximately 8–9% in precision, 10–11% in recall, and 9–10% in mAP@0.5. This reduced sensitivity to rain-induced noise suggests stronger robustness to partial occlusion, motion blur, and surface distortion.

Overall, while YOLOv7 maintains higher absolute detection accuracy under sunny conditions, YOLOv9 demonstrates superior resilience in rainy environments, retaining more consistent detection capability when conditions are most challenging. These findings indicate a clear trade-off between peak accuracy and environmental robustness, highlighting YOLOv9’s suitability for flood-monitoring scenarios where rainfall coincides with elevated flood risk and debris accumulation.

### Environmental and flood-management implications

The debris detection outputs generated by the proposed system extend beyond visual monitoring and provide actionable indicators for flood risk management and environmental decision-making. Temporal increases in debris count or density within predefined regions of interest may indicate early channel blockage, reduced water flow capacity, or higher flow resistance, which are common precursors to localized urban flooding.

The class-specific debris information further enhances interpretability. For example, the accumulation of lightweight materials such as plastic bags and Styrofoam may indicate upstream waste leakage or poor solid waste control, whereas the presence of large rigid objects (e.g., branches, tables, or canopies) are often linked to strong flow events during heavy rainfall. When combined with supporting measurements such as water level, rainfall, and flow velocity, debris data can assist in maintenance planning, targeted inspections, and early warning alerts.

From an environmental management perspective, the system enables continuous monitoring of floating pollution loads, offering data-driven support for river cleaning activities and urban waste management. Therefore, the proposed framework contributes to both real-time flood monitoring and longer-term river health assessment, supporting sustainable urban water management.

## Conclusions and future work

This study demonstrates the feasibility and effectiveness of integrating deep learning-based object detection with continuous river monitoring to improve environmental management and flood risk mitigation. By deploying YOLOv7 and YOLOv9 models at two operational river sites in Shah Alam, Malaysia, the system successfully performed real-time detection and quantification of floating debris under diverse lighting, flow, and weather conditions. YOLOv7 achieved higher overall performance, with an average mAP@0.5 of 0.918, mAP@0.5:0.95 of 0.712, precision of 0.90, and recall of 0.87, outperforming YOLOv9 by 5–12 percentage points depending on the class and evaluation metric. These results confirm that YOLOv7 provides more stable detections and fewer misclassifications for operational river environments, whereas YOLOv9 demonstrates enhanced robustness under noisy and rainy conditions.

Beyond performance improvements, this work provides several practical advancements over existing work. First, the system employs a large, diverse, multi-site dataset (15,000 images) collected from real Malaysian river environments, addressing the limitations of small or laboratory-based datasets commonly reported in prior studies. Second, the study delivers a model-level comparison between YOLOv7 and YOLOv9 under environmental variations such as rain, glare, turbidity, and background clutter, conditions rarely evaluated systematically in the literature. Third, the framework integrates real-time debris counting, density trend logging, and threshold-based alerting, transforming object detection outputs into operational indicators suitable for flood monitoring and maintenance planning.

Despite its contributions, several limitations remain. The system is still sensitive to lighting fluctuations, particularly intense glare or low-light scenarios, which can increase false detections. Submerged or semi-submerged debris remains challenging, as both YOLOv7 and YOLOv9 rely primarily on visible texture and contour cues. The detection accuracy can degrade when debris is partially occluded by waves or when flow turbulence alters object appearance. The system also requires site-specific camera placement and periodic recalibration to maintain consistent performance. Moreover, while the model generalizes well across two river sites, broader deployment may require further dataset expansion across additional river morphologies and climatic conditions.

Importantly, the debris detection outputs produced by the system have direct operational value for environmental and flood-risk management. Increases in debris count or accumulation density can act as early indicators of channel blockage, enabling timely maintenance before critical water levels are reached. Temporal debris trends support predictive maintenance and inspection planning, while class-specific detections provide insight into pollution sources and waste management issues. By converting continuous visual data into interpretable and actionable risk indicators, the proposed framework supports proactive flood mitigation and long-term river system management.

Future work will focus on integrating water-level estimation, radar or thermal modalities for low-visibility detection, and multi-site deployment across different river basins. Incorporating hydrological and meteorological data will also enable the development of a hybrid AI–hydrology early warning system, supporting city-scale flood resilience and sustainable river management.

## Data Availability

The Dataset is available upon request. Please note that the data and source codes are for academic use only.

## References

[CR1] Baharum, M. S., Awang, R. A., & Baba, N. H. (2011, June). Flood monitoring system (MyFMS). In 2011 IEEE International Conference on System Engineering and Technology; pp. 204–208. IEEE 10.1109/ICSEngT.2011.5993451.

[CR2] Chen, C., Yan, S., Yang, J., & Mei, J. (2024). An inland water detection method based on CYGNSS. *Remote Sensing Letters,**15*(1), 35–43. 10.1080/2150704X.2023.2297173

[CR3] Da Loong, J., Abdulla, R., Selvaperumal, S. K., & Rana, M. E. (2023, October). IoT-based flood monitoring system using machine learning approach, 2023 4th International Conference on Data Analytics for Business and Industry (ICDABI); pp. 47–53. 10.1109/ICDABI60145.2023.10629349.

[CR4] Faudzi, A. A. M., Raslan, M. M., & Alias, N. E. (2023, February). IoT based real-time monitoring system of rainfall and water level for flood prediction using LSTM Network. In IOP Conference Series: Earth and Environmental Science (Vol. 1143, No. 1, p. 012015). IOP Publishing. 10.1088/1755-1315/1143/1/012015.

[CR5] Hamzah, S. A., Dalimin, M. N., Md Som, M., Zainal, M. S., Ramli, K. N., Yusop, A., ... & Mustapa, M. S. (2024). Flood level detection system using ultrasonic sensor and ESP32 camera: Preliminary results. Journal of Advanced Research in Applied Mechanics, 119(1), 162–173. 10.37934/aram.119.1.162173

[CR6] Hashim, Y., Idzha, A. H. M., & Jabbar, W. A. (2018). The design and implementation of a wireless flood monitoring system. *Journal of Telecommunication, Electronic and Computer Engineering (JTEC),**10*(3–2), 7–11.

[CR7] Hassan, H., Mazlan, M. I. Q., Ibrahim, T. N. T., & Kambas, M. F. (2020, September). IOT system: Water level monitoring for flood management. In IOP Conference Series: Materials Science and Engineering; Vol. 917, No. 1, p. 012037. IOP Publishing. 10.1088/1757-899X/917/1/012037.

[CR8] He, X., Wang, J., Chen, C., & Yang, X. (2021, December). Detection of the floating objects on the water surface based on improved YOLOv5. 2021 IEEE 2nd International Conf. on Information Technology, Big Data and Artificial Intelligence (ICIBA); Vol. 2, pp. 772–777. 10.1109/ICIBA52610.2021.9688111.

[CR9] Landman, E. , “Duplicate image finder.” Accessed: Jul. 16, 2024. [Online]. Available: https://github.com/elisemercury/Duplicate-Image-Finder.

[CR10] Lee, K. F., Ng, Z. N., Tan, K. B., Balachandran, R., Chong, A. S. I., & Chan, K. Y. (2024). Artificial intelligence-integrated water level monitoring system for flood detection enhancement. *International Journal of Intelligent Systems and Applications in Engineering,**12*(19s), 336–340.

[CR11] Li, H., Yang, S., Liu, J., Yang, Y., Kadoch, M., & Liu, T. (2022a). A framework and method for surface floating object detection based on 6G networks. *Electronics,**11*(18), Article 2939. 10.3390/electronics11182939

[CR12] Li, N., Huang, H., Wang, X., Yuan, B., Liu, Y., & Xu, S. (2022b). Detection of floating garbage on water surface based on PC-Net. *Sustainability,**14*(18), Article 11729. 10.3390/su141811729

[CR13] Li, W., Jiang, R., Wu, H., Xie, J., Zhao, Y., Song, Y., & Li, F. (2023). A system dynamics model of urban rainstorm and flood resilience to achieve the sustainable development goals. *Sustainable Cities and Society,**96*, Article 104631. 10.1016/j.scs.2023.104631

[CR14] Lin, F., Hou, T., Jin, Q., & You, A. (2021). Improved YOLO based detection algorithm for floating debris in waterway. *Entropy,**23*(9), Article 1111. 10.3390/e23091111 34573736 10.3390/e23091111 PMC8465247

[CR15] Lin, T. Y., Maire, M., Belongie, S., Hays, J., Perona, P., Ramanan, D., ... & Zitnick, C. L. (2014, September). Microsoft COCO: Common objects in context. In European conference on computer vision (pp. 740–755). Cham: Springer International Publishing. 10.1007/978-3-319-10602-1_48

[CR16] Noar, N. A. Z. M., & Kamal, M. M. (2017, November). The development of smart flood monitoring system using ultrasonic sensor with Blynk applications. In 2017 IEEE 4th International Conference on Smart Instrumentation, Measurement and Application (ICSIMA) (pp. 1–6). IEEE. 10.1109/ICSIMA.2017.8312009.

[CR17] Pagatpat, J. C., Arellano, A. C., & Gerasta, O. J. (2015, April). GSM & web-based flood monitoring system. In IOP Conference Series: Materials Science and Engineering (Vol. 79, No. 1, p. 012023). IOP Publishing. 10.1088/1757-899X/79/1/012023.

[CR18] Piadeh, F., Behzadian, K., Chen, A. S., Campos, L. C., Rizzuto, J. P., & Kapelan, Z. (2023). Event-based decision support algorithm for real-time flood forecasting in urban drainage systems using machine learning modelling. *Environmental Modelling & Software,**167*, Article 105772. 10.1016/j.envsoft.2023.105772

[CR19] Rocamora, C., Puerto, H., Abadía, R., Brugarolas, M., Martínez-Carrasco, L., & Cordero, J. (2021). Floating debris in the low Segura river basin (Spain): Avoiding litter through the irrigation network. *Water (Basel),**13*(8), 1074. 10.3390/w13081074

[CR20] Saleh, A., Yuzir, A., & Abustan, I. (2020, June). Flood mapping using Sentinel-1 SAR Imagery: Case study of the November 2017 flood in Penang. In IOP Conference Series: Earth and Environmental Science (Vol. 479, No. 1, p. 012013). IOP Publishing. 10.1088/1755-1315/479/1/012013

[CR21] Shatnawi, N. (2024). Mapping floods during cloudy weather using radar satellite images. Jordan Journal of Civil Engineering, 18(1). 10.14525/JJCE.v18i1.03

[CR22] Sohn, W., Brody, S. D., Kim, J. H., & Li, M. H. (2020). How effective are drainage systems in mitigating flood losses? *Cities,**107*, Article 102917. 10.1016/j.cities.2020.102917

[CR23] Sun, X., Deng, H., Liu, G., & Deng, X. (2019). Combination of spatial and frequency domains for floating object detection on complex water surfaces. *Applied Sciences,**9*(23), Article 5220. 10.3390/app9235220

[CR24] United Nations, “The 17 Goals,” Sustainable Development. Accessed: Jul. 18, 2024. [Online]. Available: https://sdgs.un.org/goals

[CR25] Van Emmerik, T., Mellink, Y., Hauk, R., Waldschläger, K., & Schreyers, L. (2022). Rivers as plastic reservoirs. *Frontiers in Water,**3*, Article 786936. 10.3389/frwa.2021.786936

[CR26] Wang, C. Y., Bochkovskiy, A., & Liao, H. Y. M. (2022). YOLOv7: Trainable bag-of-freebies sets new state-of-the-art for real-time object detectors. arXiv. 2022 10.48550. arXiv preprint arXiv.2207.02696, 2207. 10.1109/CVPR52729.2023.00721

[CR27] Wang, C.Y., Yeh, I.H. and Mark Liao, H.Y., 2024, September. Yolov9: Learning what you want to learn using programmable gradient information. In European conference on computer vision (pp. 1–21). Cham: Springer Nature Switzerland. 10.48550/arXiv.2402.13616

[CR28] Xu, S., Tang, H., Li, J., Wang, L., Zhang, X., & Gao, H. (2023). A YOLOW algorithm of water-crossing object detection. *Applied Sciences,**13*(15), Article 8890. 10.3390/app13158890

[CR29] Zahir, S. B., Ehkan, P., Sabapathy, T., Jusoh, M., Osman, M. N., Yasin, M. N., & Jamaludin, R. (2019). Smart IoT flood monitoring system. *journal of physics: Conference series,**1339*(1), Article 012043. 10.1088/1742-6596/1339/1/012043

[CR30] Zaifudin, S. Z. S. S., Mahmud, W. M. H. W., Huong, A., Jumadi, N. A., Izaham, R. M. A. R., & Gan, H. S. (2024). Water level and flow detection system: An IoT-based flood monitoring application. *Journal of Advanced Research in Applied Mechanics,**127*(1), 89–99. 10.37934/aram.127.1.8999

[CR31] Zain, N. M., Elias, L. S., Paidi, Z., & Othman, M. (2020). Flood warning and monitoring system (FWMS) using GSM technology. *Journal of Computing Research and Innovation,**5*(1), 8–19. 10.24191/jcrinn.v5i1.158

[CR32] Zakaria, M. I., Jabbar, W. A., & Sulaiman, N. (2023). Development of a smart sensing unit for LoRaWAN-based IoT flood monitoring and warning system in catchment areas. *Internet of Things and Cyber-Physical Systems,**3*, 249–261. 10.1016/j.iotcps.2023.04.005

[CR33] Zayed, M., & Saleh, M. (2025). Assessing the backwater rise of floating barriers with racks for various blockages in open channels. *Journal of Engineering and Applied Science*, SpringerOpen. 10.1186/s44147-025-00593-0

[CR34] Zhang, L., Zhang, Y., Zhang, Z., Shen, J., & Wang, H. (2019). Real-time water surface object detection based on improved faster R-CNN. *Sensors,**19*(16), Article 3523. 10.3390/s19163523 31408971 10.3390/s19163523 PMC6719926

[CR35] Zhang, L., Wei, Y., Wang, H., Shao, Y., & Shen, J. (2021). Real-time detection of river surface floating object based on improved refinedet. *IEEE Access,**9*, 81147–81160. 10.1109/ACCESS.2021.3085348

[CR36] Zhang, L., Xie, Z., Xu, M., Zhang, Y., & Wang, G. (2023). EYOLOv3: An efficient real-time detection model for floating object on river. *Applied Sciences,**13*(4), Article 2303. 10.3390/app13042303

[CR37] Zhou, Z., Sun, J., Yu, J., Liu, K., Duan, J., Chen, L., & Chen, C. P. (2021). An image-based benchmark dataset and a novel object detector for water surface object detection. *Frontiers in Neurorobotics,**15*, Article 723336. 10.3389/fnbot.2021.723336 34630064 10.3389/fnbot.2021.723336 PMC8497741

